# The EFF-1A Cytoplasmic Domain Influences Hypodermal Cell Fusions in *C*. *elegans* But Is Not Dependent on 14-3-3 Proteins

**DOI:** 10.1371/journal.pone.0146874

**Published:** 2016-01-22

**Authors:** Jessica H. Shinn-Thomas, Jacob J. del Campo, Jianjun Wang, William A. Mohler

**Affiliations:** Department of Genetics and Developmental Biology, University of Connecticut Health Center, MC-6403, 263 Farmington Avenue, Farmington, CT 06030–6403, United States of America; Institut de Génétique et Développement de Rennes, FRANCE

## Abstract

**Background:**

Regulatory and biophysical mechanisms of cell-cell fusion are largely unknown despite the fundamental requirement for fused cells in eukaryotic development. Only two cellular fusogens that are not of clear recent viral origin have been identified to date, both in nematodes. One of these, EFF-1, is necessary for most cell fusions in *Caenorhabditis elegans*. Unregulated EFF-1 expression causes lethality due to ectopic fusion between cells not developmentally programmed to fuse, highlighting the necessity of tight fusogen regulation for proper development. Identifying factors that regulate EFF-1 and its paralog AFF-1 could lead to discovery of molecular mechanisms that control cell fusion upstream of the action of a membrane fusogen. Bioinformatic analysis of the EFF-1A isoform’s predicted cytoplasmic domain (endodomain) previously revealed two motifs that have high probabilities of interacting with 14-3-3 proteins when phosphorylated. Mutation of predicted phosphorylation sites within these motifs caused measurable loss of *eff-1* gene function in cell fusion *in vivo*. Moreover, a human 14-3-3 isoform bound to EFF-1::GFP *in vitro*. We hypothesized that the two 14-3-3 proteins in *C*. *elegans*, PAR-5 and FTT-2, may regulate either localization or fusion-inducing activity of EFF-1.

**Methodology/Principal Findings:**

Timing of fusion events was slightly but significantly delayed in animals unable to produce full-length EFF-1A. Yet, mutagenesis and live imaging showed that phosphoserines in putative 14-3-3 binding sites are not essential for EFF-1::GFP accumulation at the membrane contact between fusion partner cells. Moreover, although the EFF-1A endodomain was required for normal rates of *eff-1*-dependent epidermal cell fusions, reduced levels of FTT-2 and PAR-5 did not visibly affect the function of wild-type EFF-1 in the hypodermis.

**Conclusions/Significance:**

Deletion of the EFF-1A endodomain noticeably affects the timing of hypodermal cell fusions *in vivo*. However, prohibiting phosphorylation of candidate 14-3-3-binding sites does not impact localization of the fusogen. Hypodermal membrane fusion activity persists when 14-3-3 expression levels are reduced.

## Introduction

Intercellular fusion is a crucial biological process in the development of many organisms. In humans, it allows for fertilization and the functional development of muscle, bone, placental tissue, and the lens of the eye [1–8]. Ongoing stem cell, regenerative, and cancer research implicates cell fusion events in natural and experimental cellular reprogramming events, as well as in the progression of cancer cells towards malignancy [9–18]. Other examples of cell fusion are seen in the biology of organisms important to crop agriculture, food processing, and infectious disease [5,7,19–25].

Despite its prevalence and significance, understanding the molecular, biochemical, and biophysical mechanisms of developmental cell fusions is in its infancy [3,5,7,26]. The molecular mechanisms of virus-cell membrane fusion and intracellular membrane fusion are much better understood than cell-cell fusion [26–29], and that knowledge has already yielded crucial medicines that work via direct modulation of fusogenic reactions during infection [30–32]. Detailed understanding of the mechanism of cell-cell fusion in animals could be crucial for the development of many possible technologies and therapies, including tissue engineering and regeneration, cancer immunotherapy, and design of therapies for fusion abnormalities affecting muscle, bone, and reproduction [3].

The nematode *Caenorhabditis elegans (C*. *elegans*) is an ideal organism in which to study cell fusion because of its nearly invariant sequence of embryonic and postembryonic fusion events, wherein over one-third of somatic cells fuse to develop multiple syncytial cells in major tissues such as the vulva, pharynx and hypodermis (epidermis) [33–35]. Until recent years, no molecule was known to act as an intercellular fusion protein during the developmentally programmed formation of these multinucleated tissues. It is now known that two paralogous “FF” proteins found in *C*. *elegans*, EFF-1 and AFF-1 (epithelial-fusion-failure and anchor cell-fusion-failure), are necessary and sufficient to fuse complementary sets of neighboring cells into multinucleated structures [36–42].

Significantly, EFF-1::GFP (which has detectable, but only partial, *Eff* rescuing activity) becomes concentrated at the borders of fusion-fated cells in the developing embryonic hypodermis immediately before fusion occurs [38]. Discrete localization to fusion competent cell-cell borders has also been observed in SF9 and S2R+ insect cells, which fuse when induced to express nematode EFF-1 [41,43]. While polarization and localization of cellular fusogens is important for the proper patterning of tissues, the molecular mechanism behind this tightly regulated selection remains unknown. The importance of such control, however, has been demonstrated *in vivo*, as unregulated expression of EFF-1 or AFF-1 leads to a lethal phenotype, in which promiscuous and destructive cell-cell fusion occurs throughout and between tissues [37,38,40].

Many questions remain unanswered regarding how EFF-1 is regulated. How is EFF-1 signaled to specifically localize to the membrane border between fusion-fated cells prior to fusions? What constrains EFF-1 from accumulating at the membrane contact with a cell neighbor that is not a fusion partner? Once localized to the membrane interface, how is EFF-1 triggered to actuate the fusion between membrane bilayers? Identifying factors that interact with EFF-1 to control its activity and localization patterns will contribute to a better understanding of the general mechanism of cell fusion mediated by FF proteins, and fusogens to be discovered in other systems.

Sequence analysis of FF proteins shows FF members in other nematode species in addition to a small number of arthropods and a ctenophore, chordate, and protist [27,40,42,44]. Recent data shows structural homology between EFF-1’s ectodomain and class II viral fusion proteins; however, the mechanism by which EFF-1 fuses membranes is different from class II viral fusogen mechanisms [45,46]. Therefore, we focused our studies on the FF protein domains’ structure and function. EFF-1 has four alternatively spliced isoforms, of which only two, EFF-1A and EFF-1B, have a transmembrane anchor. EFF-1A and EFF-1B share similar extracellular and transmembrane domains; however, EFF-1B’s C-terminal cytoplasmic domain is shorter than EFF-1A’s and varies greatly in its sequence [36]. Three lines of evidence indicate that EFF-1A functions more potently as a developmental fusogen than does EFF-1B, and that much of this increased potency in encoded in the EFF-1A C-terminus. First, EFF-1A cDNA rescues *eff-1(oj55)* mutant defects in *C*. *elegans*, while EFF-1B cannot rescue these mutants [38]. Second, Podbilewicz *et al*. showed that both EFF-1A and EFF-1B localize to the cell surface and fuse transfected SF9 insect cells in culture [41]. However, a large amount of EFF-1B cDNA, five times higher than that used for EFF-1A, was required in order to achieve similar cell surface expression levels and multinucleation. These results suggest that much more EFF-1B mRNA must be translated for an equivalent concentration of fusogen to be activated and function at the plasma membrane. Third, Sapir *et al*. reported that replacing AFF-1’s native transmembrane domain and C-terminal cytoplasmic domain with those of EFF-1A increased cell surface expression and multinucleation in SF9 cells [40]. These results all support the hypothesis that EFF-1’s cytoplasmic domain has a functional role in contributing to efficient and potent cell fusions, possibly by increasing surface expression, maintaining EFF-1 membrane stability, or triggering EFF-1 to actuate fusion. Interestingly, cytosolic C-terminal truncation of some viral fusogens greatly reduces or eliminates fusogenic activity, and cell surface expression [47–50]. Thus, intracellular sequences appear to be important to the function of a broad range of unrelated fusogenic proteins.

We previously used a web-based application, Minimotif Miner (MnM 1.0), to search within EFF-1’s cytoplasmic domain for instances of short peptide motifs (<15 residues) of known function [51–53]. This analysis revealed two candidate 14-3-3-binding motifs as the highest scoring potentially functional sites. 14-3-3 proteins are highly conserved, eukaryotic proteins that bind phosphorylated motifs within a broad spectrum of interacting target proteins [54–57].

Many of the known actions of 14-3-3 family members upon their binding partners correspond to notable aspects of EFF-1 function *in vivo*. 14-3-3 proteins are often referred to as “scaffolds” that bring proteins into proximity with each other by virtue of their rigidity and propensity to form dimers [55,58]. Multiple studies have demonstrated a role for 14-3-3s in asymmetrically restricting cell polarity determinants in *C*. *elegans* zygotes, mammalian epithelia, and *Drosophila* oocytes and epithelial cells [59–63]. Evidence also implicates 14-3-3 proteins in facilitating forward transport of plasma membrane proteins through the secretory pathway [56,64,65]. 14-3-3 proteins have been shown to mask membrane protein translocation signals, such that release from 14-3-3-binding enhances membrane protein expression. Conversely, 14-3-3 binding to a cell surface protein could mask ER retention signals, thereby releasing the protein from the ER. 14-3-3 dimers are also modeled to initiate transport from the ER through the secretory pathway by simultaneously binding both to a membrane-bound protein and either to forward transport accessory protein complexes or to proteins that inactivate ER retention machinery. Based on these various models of action by 14-3-3s, we hypothesized that 14-3-3s could regulate EFF-1 delivery, accumulation, or activation at the fusion-fated cell surface.

Subsequent experiments suggested that 14-3-3 proteins might be required for timely embryonic hypodermal cell fusions in *C*. *elegans*. First, mutation of the candidate 14-3-3-binding motifs in EFF-1A caused loss of the *Eff*-phenotype-rescuing activity of an *eff-1* transgene tested *in vivo*. Second, human 14-3-3η bound to EFF-1A::GFP when both proteins were co-expressed in mammalian cells [51]. Based on these findings, we hypothesized that 14-3-3 proteins specifically regulate EFF-1-mediated cell fusion events *in vivo*, perhaps by governing EFF-1’s translocation from internal organelles to the cell surface, by anchoring and/or clustering EFF-1A at the plasma membrane border programmed to fuse, or by effecting an activating change in the conformation of EFF-1A that drives membrane fusion. EFF-1’s ectodomain shows structural homology to class II viral fusion proteins and a unique cell-cell fusion mechanism from class II viral fusogens for which the trigger of EFF-1 mediated cell fusion is still unknown [45,46]. Intracellular factors, such as 14-3-3, that are predicted to bind to EFF-1’s cytoplasmic domain could act as the “trigger” for fusion initiation.

In this paper, we further investigate the role of the only known *C*. *elegans* 14-3-3 proteins, PAR-5 and FTT-2 [66], in controlling EFF-1’s spatiotemporal function and localization. We confirm, through analysis of endogenous mutations, that the C-terminal tail of EFF-1A is required for the precise timing of cell fusions in the embryo. However, we find that normal expression of 14-3-3 proteins is not essential for EFF-1-induced hypodermal cell fusions. Furthermore, the candidate 14-3-3-binding sites within EFF-1A are not required for timely localization of EFF-1A::GFP to fusion-competent hypodermal cell contacts. These new results combine with previously published findings to indicate that potentiation of EFF-1 function and localization in the hypodermis by these putative C-terminal phospho-motifs does not require interaction with normal levels of 14-3-3 proteins. However, fusion activity is noticeably enhanced by presence of the EFF-1A C-terminal cytoplasmic tail.

## Materials and Methods

### Strains

Unless otherwise indicated, all *C*. *elegans* strains were cultured according to standard techniques [67] at 20°C on Nematode Growth Media (NGM) agar plates supplemented with 200 μg/ml streptomycin sulfate and a streptomycin-resistant strain of *Escherichia coli (E*. *coli)*, OP50-1 (*Caenorhabditis* Genetics Center, St. Paul, MN).

SU93 (*jcIsI* [*AJM-1*::*GFP*, *rol6(su1006)*, *unc29(+)*] *IV*): A kind gift from Jeff Simske [68,69].

FC57 (*eff-1(zz1) mIs12 II*): This strain was isolated in a forward genetic non-complementation screen for new *eff-1* alleles. Strain CB5584 (*mIs12*[*myo-2*::*GFP*, *pes-10*::*GFP*, *gut*::*GFP*] *II*) has a pharynx-specific GFP transgene integrated within ~1cM of *eff-1* on Chromosome II. CB5584 males were mutagenized with EMS as described in [67]. Mutated males were crossed with FC60 (*eff-1(oj55) II; unc-119 (e2498) III*; *jcIsI*[*AJM-1*::*GFP*, *rol6(su1006)*, *unc29(+)*] *IV*) hermaphrodites and the subsequent F1 generation was screened for transheterozygote progeny (new allele/*oj55*) with Eff mutant phenotypes. Worms were made homozygous by selecting for the *mIs12* marker and backcrossed three times to N2 (Bristol) [38].

FC70 (*eff-1(zz7) II; jcIs1 IV*): FC70 was generated in the same fashion as FC57, by isolation of alleles from a non-complementation screen over *eff-1(oj55)*. All other *eff-1* alleles used here were reported in [36,38].

FC75 (*eff-1(zz10) II; jcIs1 IV*): FC75 was generated in the same fashion as FC57. This allele generates a null mutation (W7stop).

FC80 (*eff-1(zz1) mls12 II; jcIs1 IV*): FC57 (*eff-1(zz1) mIs12 II*) was crossed to SU93 (*jcIs1 IV*), and progeny homozygous for both fluorescence reporters were isolated.

FC183: (*zzIs22*[*pJdC41(EFF-1*::*GFP)*, *pRF4(rol-6(su1006))*]) [38]

FC254 (*unc-119 (e2498) III; zzEx98*[*EFF-1(S632/634/654A)*::*GFP*, *unc-119(+)*]): Site-directed mutagenesis (QuikChange II XL Kit, Stratagene) in EFF-1::GFP (pJdC41) was used to mutate three predicted phosphorylatable serine residues in both 14-3-3-consensus sites to alanines. Mutations were introduced in two consecutive steps. First, primers were designed to mutate S654 to S654A in pJdC41 (Forward primer: 5’GCGGCACTATAGCGCTAGCCAGTACATTCCGCGG, Reverse primer: 5’CCGCGGAATGTACTGGCTAGCGCTATAGTGCCGC). The resulting construct, EFF-1(S654A)::GFP (pJHS16), was used as a template to create EFF-1(S632/634/654A)::GFP (pJHS18) by mutating the remaining serines to alanine, S632/634A (Forward primer: 5’GGTGCAAGAGCTAGTGCCGAGCCCCACG, Reverse primer: 5’CGTGGGGCTCGGCACTAGCTCTTGCACC). By traditional *C*. *elegans* microinjection techniques, pJHS18 was co-injected with an *unc-119(+)* rescuing transgene (pDPmm016, a kind gift from Jeffrey Simske) into a severely paralyzed Unc strain, CB4845 (*unc-119 (e2498) III*, obtained from David Pilgram). Transgenic worms were maintained by identifying non-Unc worms that expressed EFF-1(S632/634/654A)::GFP.

HC396 (*unc-119 (e2498) III; qtIs19*[*elt-3p*::*yfp*, *unc-119+*]: Expresses a cytoplasmic fluorescent protein reporter in a subset of hypodermal precursor cells in the developing embryo (a kind gift from Craig Hunter, [70].)

FC275 (*ftt-2 (n4426Δ) X; unc-119 (e2498) III; qtIs19*[*elt-3p*::*yfp*, *unc-119*+]): A *C*. *elegans* strain that contains a null allele of *ftt-2*, MT14355 (*ftt-2 (n4426Δ) X*), was generously provided by Robert Horvitz’s lab. This deletion allele removes the *ftt-2* promoter and start codon. MT14355 is characterized by a “bagging” phenotype in which embryos are retained and hatch *in utero* (a few embryos are layed before bagging is observed) [71]. MT14355 hermaphrodites were crossed with HC396 heterozygous males (*unc-119 (e2498) III; qtIs19*[*elt-3p*::*yfp*, *unc-119+*]. Worms homozygous for *ftt-2 (n4426Δ)* and *elt-3p*::*yfp* were isolated by identifying “bagging” non-Unc hermaphrodites that produced 100% *elt-3p*::*yfp-*expressing progeny. This new strain was genotyped to confirm homozygosity of the *ftt-2* deletion allele using a primer set (Forward primer: 5’TGAGAAAGAGAAGAAAGAGGGCG, Reverse primer: 5’GATAGGGAGAGACGCACAGAAAAC) that amplifies only the wild-type allele. Individual worms were lysed in 3 μl of single-worm lysis buffer (50 mM KCl, 10 mM Tris pH 8.3, 2.5 mM MgCl_2_•6 H_2_O, 0.45% NP-40, 0.45% Tween-20, 0.01% Gelatin, plus 1 mg/ml Proteinase K) by freezing the worms in lysis buffer at -80°C for 1 hour, heating to 65°C for 1.5 hours, and finishing with a 15 minute incubation at 95°C. The presence or absence of the wild-type allele in *ftt-2* was detected by PCR using *Taq* DNA polymerase (Invitrogen, #10342). No wild-type allele was detected in strain FC275.

FC276 (*ftt-2 (n4426Δ) X; jcIs1 IV*): MT14355 (*ftt-2 (n4426Δ) X*) heterozygous males were crossed with SU93 (*jcIs1 IV*) hermaphrodites. Worms homozygous for both loci were obtained and genotyped in a manner similar to FC275.

FC279 (*par-5(it55) unc-22(e66) IV/nT1*[*unc-*?*(n754dm) let-*?] *(IV;V); zzEx116*[*AJM-1*::*ceCherry)*, *lbp-1p*::*gfp*]): Homozygous loss-of-function *par-5* mutants are maternal-effect lethal and therefore cannot be propagated [63]. However, a balanced heterozygous strain, KK299 (*par-5(it55) unc-22(e66) IV/nT1*[*unc-*?*(n754dm) let-*?] *(IV;V)*), containing a balanced strong loss-of-function *par-5* allele was obtained from the *Caenorhabditis* Genetics Center (CGC) [63]. This strain produces two classes of viable offspring: fertile heterozygotes that are Unc, and *par-5 unc-22* homozygotes that twitch and lay only dead eggs. However, the balanced heterozygous strain is highly unstable, preventing efficient genetic crosses with reporter strains. Consequently, we co-injected the fluorescence-tagged constructs *lbp-1p*::*gfp* (pKK1) and AJM-1::ceCherry (pJS555, a generous gift from Jeffrey Simske) into the balanced strain, and the resulting balanced, transgenic strain was maintained. Twitching, unbalanced offspring of this strain—homozygous for *par-5 (it55) unc-22(e66)* and expressing the transgene array—were identified and used for imaging of the Par mutant phenotype.

FC280 (*eff-1(zz10) II; unc-119 (e2498) III; qtIs19*[*elt-3p*::*yfp*, *unc-119+*]): FC142 (*eff-1(zz10) II*) hermaphrodites were crossed with HC396 heterozygous males (*unc-119 (e2498) III; qtIs19*[*elt-3p*::*yfp*, *unc-119+*]). “Dumpy” worms (a phenotype of homozygous *eff-1(zz10)* animals) that produced 100% *elt-3p*::*yfp* positive progeny were isolated to create the homozygous strain.

### RNAi

A *par-5* RNAi feeding construct was generously provided by Sieu Sylvia Lee’s lab [72] and was transformed into the *E*. *coli* feeding strain HT115 (DE3) [73]. The transformed bacteria were streaked from a frozen stock onto a Luria Broth (LB)-Amp agar plate overnight at 37°C for 16 hours. One colony of *par-5* RNAi was grown in 5 ml LB-Amp media overnight at 37°C for 16 hours. Sixty-millimeter NGM plates supplemented with 2 mM IPTG were seeded with 200 μl of *par-5* RNAi overnight culture, a sufficient quantity to sustain multiple generations of propagating *C*. *elegans*, and the plated bacteria were allowed to induce siRNA expression for 24 hours at room temperature. Five L2/L3-stage FC275 larvae (P0) were washed twice in a 200 μL drop of M9 buffer then transferred to the induced *par-5* RNAi NGM plate. Larvae were cultured at 20°C and monitored for hatching and growth of their offspring (F1) to the L3/L4 stage before the F1-L3/L4 larvae were mounted for imaging (see below).

### EFF-1 Localization Detection

Transgenic embryos from strain FC254 were obtained by standard dissection from non-Unc gravid adults and were mounted in Egg Buffer (118mM NaCl, 48mM KCl, 3mM CaCl, 3mM MgCl, 5mM Hepes pH7.0) with 1% methyl cellulose (Sigma-Aldrich, #274429) and 0.09% 20-μm polystyrene beads (a 1:30 dilution from stock suspension, Polysciences, Inc., #18329). Time-lapse, widefield fluorescence microscopy (PlanApo 60X, 1.4 NA, Nikon Eclipse TE300 with automatic shutter control, Cooke Sensicam cooled CCD, Metamorph acquisition control software) was used to record the localization of EFF-1(S632/634/654A)::GFP in embryos. Eight one-micron-spaced optical sections through the dorsal or ventral hypodermal surface were imaged every 2.5 minutes for 15 timepoints. Maximum intensity Z-projections of four (ventral) or five sections (dorsal) were rendered using ImageJ [74]. FC183 embryos were imaged using the techniques described under “Monitoring Cell-Cell Fusion”.

### Monitoring Cell-Cell Fusion

Embryos from SU93, FC75, HC396, FC280, FC80, FC275, FC276, and FC279 were isolated and mounted as above. A spinning disk confocal microscope (PlanFluor 40X, NA 1.30 objective, Perkin Elmer/Prairie Ultraview RS5 laser launch system, Yokogawa CSU 21 microlens scanhead, Nikon Eclipse TE2000-E inverted microscope, Hamamatsu ORCA-AG cooled CCD camera) fitted with a Perfect Focus System (Nikon) was controlled by MetaMorph software (Molecular Devices) to create 4-dimensional (4D) renditions of cell fusions during embryonic development. YFP signal was excited with a 514 nm laser or GFP signal was excited with a 488 nm laser. Stacks of confocal optical sections spaced 1μm apart through the whole embryo were collected every 2.5 minutes for 12 hours. Maximum intensity Z-projections of the confocal stacks over the 12-hour time period were rendered using MetaMorph or ImageJ [74]. (For HC396 and FC80, images were taken without the Perfect Focus System.)

Larvae from FC275 (*par-5* RNAi), HC396 and FC280 were transferred to a 200 μL drop of M9 buffer (22mM KH_2_PO_4_, 42mM Na_2_HPO_4_, 86mM NaCl) containing 0.1 M levamisole to paralyze the worms for imaging purposes. Larvae were mounted between a slide and coverslip in M9 buffer with 0.1 M levamisole, 1% methyl cellulose, 0.09% 20-μm polystyrene beads (Polysciences, Inc., #18329) and imaged using spinning disk confocal microscopy (Plan Fluor 20X, NA 0.50 objective). YFP signal was excited with a 514 nm laser. Confocal stacks of optical sections spaced 1μm apart through the whole larva were collected. Maximum intensity Z-projections from sections through the hypodermis were rendered using ImageJ [74].

## Results

### EFF-1’s Cytoplasmic Domain Is Required for Normal Cell Fusion Activity *in vivo*

**Fig 1:**
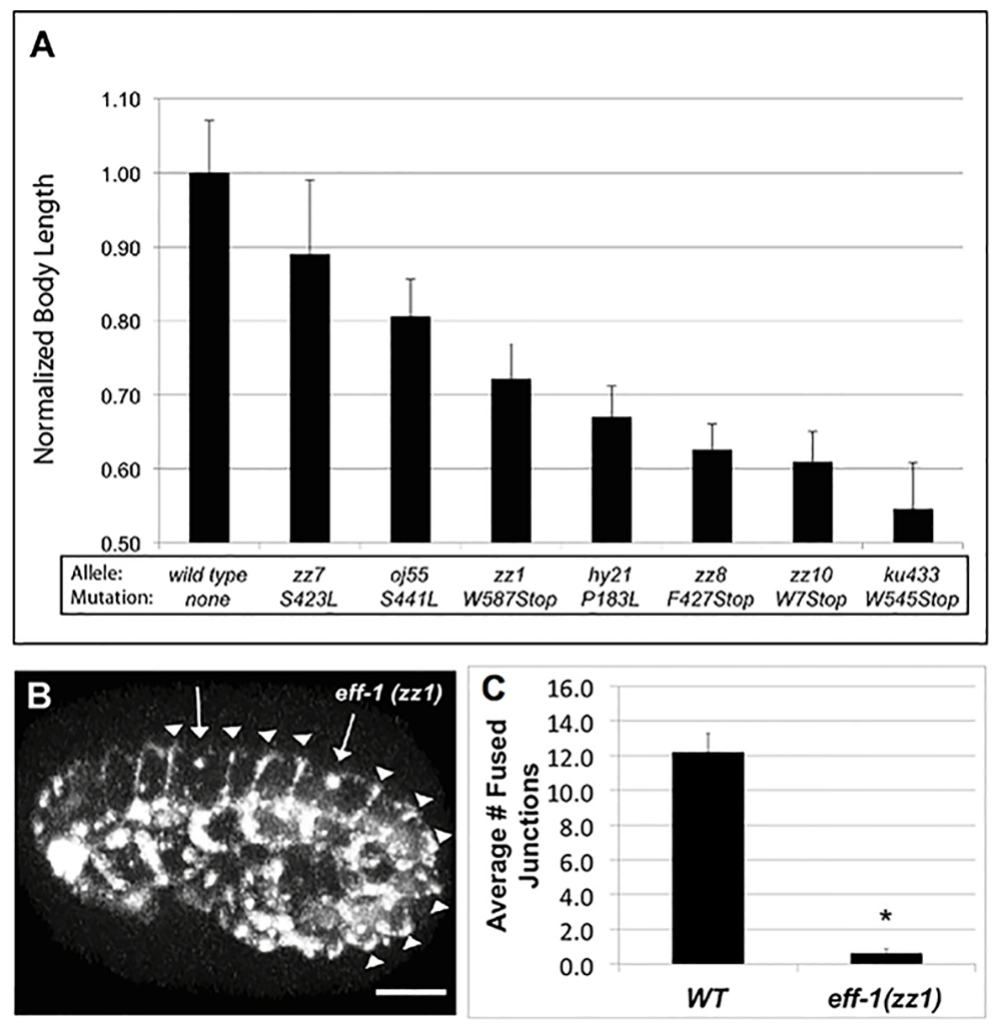
*eff-1(zz1)* mutants display a growth defect and are delayed in embryonic hyp7 cell fusions. (A) Normalized body-length measurements from 96-hour-old adult worms carrying distinct mutations in *eff-1*. Genotype-matched control strains used for each normalization were: N2 (Bristol) for alleles *oj55*, *hy21*, *zz10*, and *ku433*; C55584 (*mIs12 II)* for *zz1* and *zz8*; SU93 (*jcIs1)* for *zz7*. Mean and standard deviation are shown (n = 17–76). (B) Confocal volume projection of a 1.5-fold *eff-1(zz1)* mutant embryo expressing AJM-1::GFP. Arrows show sites in hyp7 where AJM-1::GFP has disappeared, indicating the final stages of fusion between 2 pairs of cells. Arrowheads show intact cell junctions between hyp7 cells that are fusion-delayed. A wild-type AJM-1::GFP embryo at an equivalent embryonic stage is shown in Fig 4 (t = 450) where arrows show fused junctions. Anterior is left and dorsal is up. Scalebar = 10 μm. (C) Average number of fused hyp7 cell borders seen before the beginning of embryonic movement (~1.5 fold stage) in wild-type (n = 5) and *eff-1(zz1)* (n = 13) embryos. Error bars show standard error of the mean. *p<0.001 in an independent t-test.

We have briefly discussed and characterized the *eff-1(zz1)* mutant elsewhere [51]. This mutant has a premature Trp>Stop nonsense mutation at residue 587 that eliminates all but two residues of EFF-1A’s cytoplasmic domain (S1 Fig). Nonsense mediated decay (NMD) analysis suggests that the loss of function in *eff-1(zz1)* animals is due to defects within the expressed protein, rather than message instability brought on by the nonsense mutation (see S1 Supporting Information). To more fully understand the contribution of the missing domain to normal function, we extended our analysis of the *eff-1(zz1)* phenotype. All *eff-1(zz1)* mutant hatchlings exhibited abnormal tail whip morphology visible using a dissecting microscope. This phenotype is also manifest in every other known *eff-1* loss-of-function allele. In order to more precisely appraise the severity of this molecular defect, we compared other morphological measures of the Eff phenotype among a collection of alleles. We found that *eff-1(zz1)* animals have a rather normal-looking body morphology, in striking contrast to strong loss-of-function *eff-1* mutants (e.g. *zz8*, *zz10*, and *ku433* alleles), which are Dumpy. Quantitative comparison of adult (96 hr) body length also showed an intermediate defect in *eff-1(zz1)* when compared to both mild and null alleles of *eff-1* (Fig 1A). These phenotypic characteristics suggest that *eff-1(zz1)* mutants have sufficient functional EFF-1 activity to execute cell fusions required for most of normal morphogenesis, but that some fusions are sufficiently delayed to reveal fully penetrant defects in the larval tail whip and adult body length. Alternatively, other non-fusion-related events might be affected.

To gain further insight into the defects induced by this mutation, we directly monitored the timing of embryonic fusion events in the hypodermis of *eff-1(zz1)* mutant embryos by observing AJM-1::GFP at intercellular junctions via high-resolution imaging. By the 1.5- to 2-fold stage of normal embryonic development, 17 dorsal hypodermal cells (comprising 16 membrane contacts) normally complete fusion as they begin forming the hyp7 syncytium [33,75]. In strong *eff-1* mutant embryos, none of these cell junctions disappear (refer to Fig 4 for AJM-1::GFP control images). However, in *eff-1(zz1)* embryos, we did observe disappearance of some hypodermal cell junctions–a phenomenon associated with completion of cell fusion events—within this normal timeframe (Fig 1B, S1 Movie). We more precisely quantified the timing of these fusions relative to other contemporaneous developmental events. To compare the activity of EFF-1 expressed from *eff-1(zz1)* against the activity from the wild-type gene (Fig 4, t = 450), we counted the number of fused dorsal hyp7 junctions before the first embryonic movement at the 1.5-fold stage. This test revealed a significant difference between *eff-1(zz1)* embryos and wild-type embryos in the number of fusion events completed by this stage (p<0.001 in independent t-test, Fig 1C).

### Removal of Predicted 14-3-3 Binding Motif Phosphorylation Sites Does Not Disrupt Normal EFF-1 Hypodermal Localization

Previous results demonstrated that EFF-1 requires the serine residues within both of the two potential 14-3-3-binding phospho-motifs (S1 Fig) in order for an *eff-1* full-length transgene to induce timely fusions *in vivo*. Mutation of these serines to alanine, a residue incapable of phosphate modification, prevented rescue of cell fusions when EFF-1(S632/634A) and EFF-1(S654A) were expressed transgenically in an *eff-1(oj55)* mutant background. Moreover, human 14-3-3η binding to EFF-1::GFP *in vitro* was reduced or eliminated following these serine-to-alanine substitutions, strongly suggesting that 14-3-3 proteins might engage EFF-1 at these sites *in vivo* [51].

**Fig 2:**
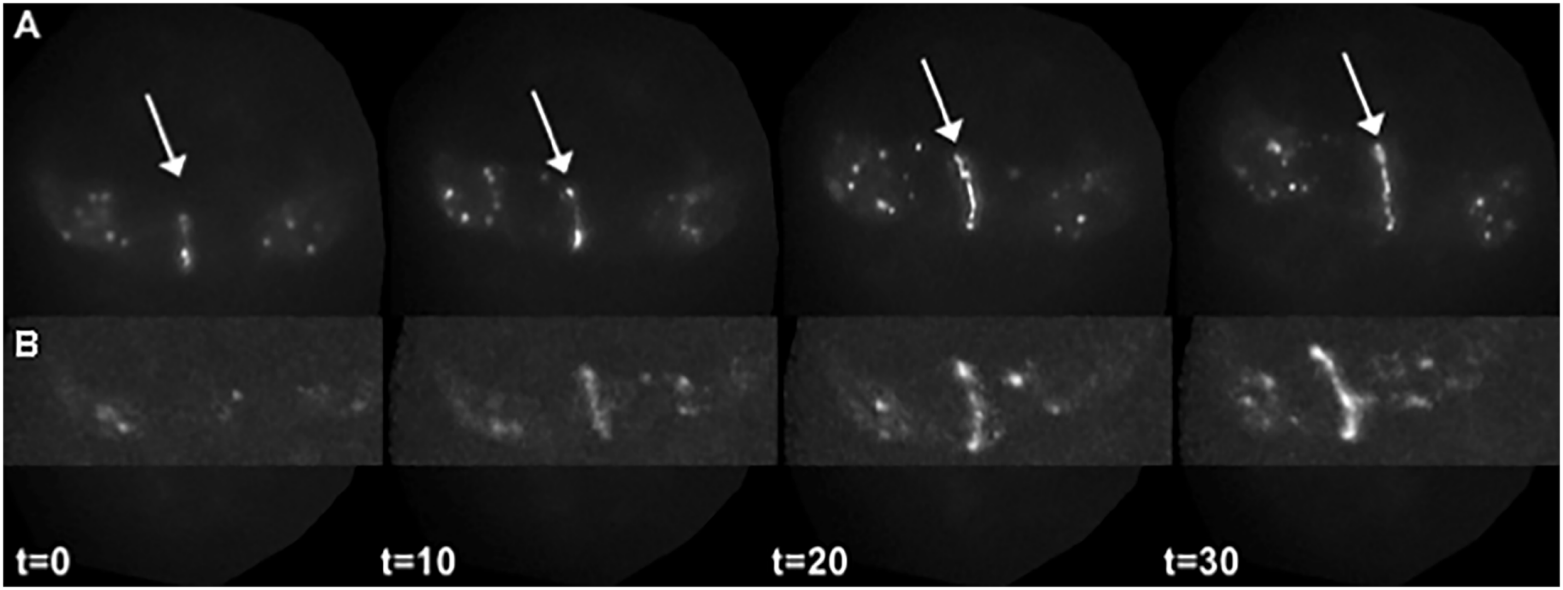
EFF-1(S632/634/654A)::GFP localization patterns are similar to wild-type EFF-1::GFP *in vivo*. Time-lapse images of EFF-1(S632/634/654A)::GFP show spatiotemporal localization to fusion competent ventral (A) cell borders (arrow) in the absence of phosphorylatable residues within putative 14-3-3 binding sites compared to wild-type EFF-1::GFP (B). One-micron-spaced image stacks were captured every 2.5 minutes using widefield (A) or confocal (B) microscopy, and maximum intensity Z-projections of the ventral surface were rendered. In 100% of the mutant embryos (n = 3), the same pattern of junctional localization is seen as for wild-type EFF-1::GFP (B) [38]. Anterior is up and posterior is down. Times shown are in minutes.

We hypothesized that the predicted 14-3-3 binding motifs are required for EFF-1::GFP localization to characteristic fusion-competent hypodermal cell contacts *in vivo* [38]. To test this, we mutated all three serines in both 14-3-3-binding sites (EFF-1(S632/634/654A)::GFP) to prevent phosphorylation and possible binding of 14-3-3 to these putative phospho-motifs within EFF-1::GFP. Projections of widefield epifluorescence stacks in Fig 2A show the localization pattern of EFF-1(S632/634/654A)::GFP between a pair of hypodermal cells programmed to fuse on the ventral side of the embryo (Fig 2A and S2 Movie). When compared to wild-type EFF-1::GFP (Fig 2B) [38], the mutant EFF-1(S632/634/654A)::GFP localized and accumulated to the junction between the ventral cells with normal timing during hypodermal enclosure and elongation. Similar results were observed with EFF-1(S632/634/654A)::GFP localization between hypodermal cells programmed to fuse on the dorsal side of the embryo (S3 Movie). No aberrant or ectopic localization patterns were observed compared to wild-type EFF-1::GFP [38].

### Normal Levels of FTT-2 or PAR-5 Are Not Required for Hypodermal Cell Fusions in EFF-1-Expressing Cells

**Fig 3:**
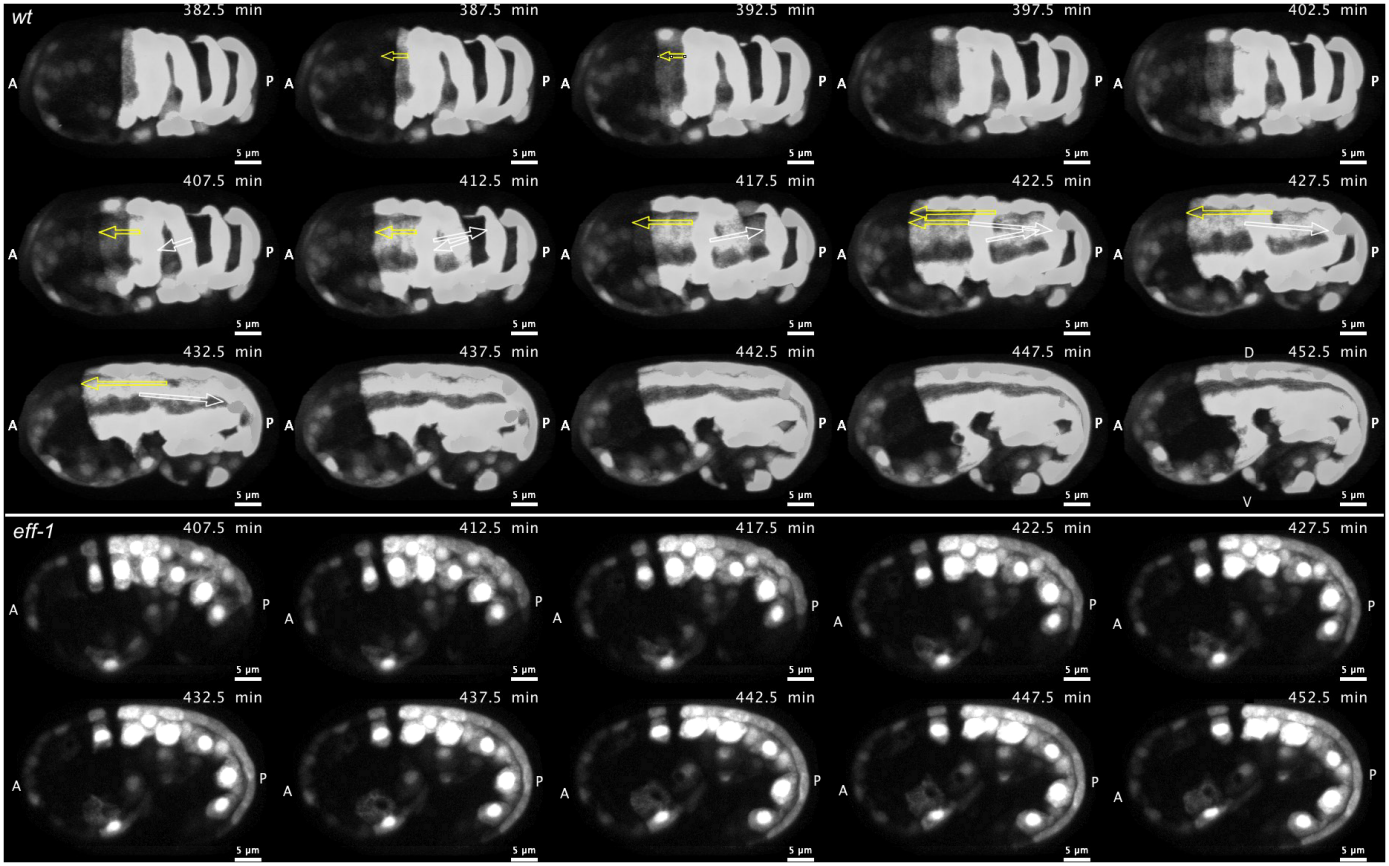
*elt-3p*::*yfp* reporter of cell-cell fusions. Time-lapse images of a wild-type embryo (top panel) expressing the *elt-3p*::*yfp* cytoplasmic hypodermis-specific reporter show cell-cell fusions in the developing epidermis. Fusion pores are revealed by diffusion of YFP from labeled cells to neighboring unlabeled cells. Both white and yellow arrows denote the anterior and posterior limits of each successively expanded multinucleated cell during the stepwise fusion events that create the large hyp6 and hyp7 syncytia. Yellow arrows show specific fusion events that were monitored in optical-section time-lapse recordings of mutant and rescued genotypes in Figs 5 and 7. The *elt-3p*::*yfp* pattern of cytoplasmic and nuclear fluorescence in the dorsal hypodermis of the *eff-1* mutant (bottom panel) remains variegated throughout embryonic elongation. Images are maximum intensity projections of 27 one-micron-spaced confocal optical sections through the entire embryo shown at 5-minute intervals. Scalebar = 5 μm.

**Fig 4:**
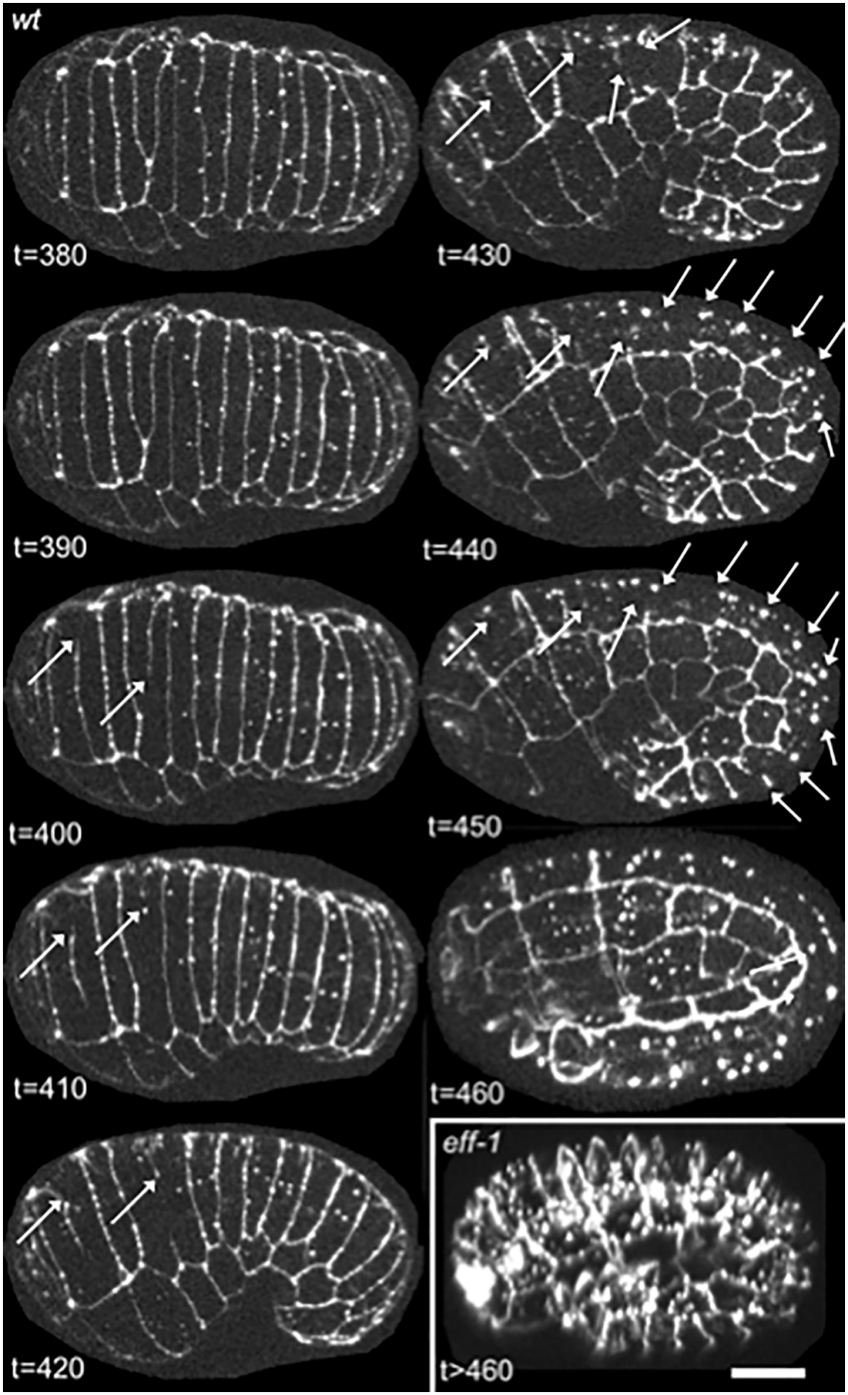
AJM-1::GFP reporter of cell-cell fusions. Time-lapse images of a wild-type embryo expressing a sub-adherens junction marker, AJM-1::GFP, show the disappearance of borders between fused cells (arrows). An *eff-1* mutant embryo (inset) shows no cell fusions at a timepoint past that at which most fusions are completed in wild-type embryos. Anterior is left and dorsal is facing the viewer (t = 380–410) or oriented up (t = 420–460). Images shown are maximum intensity Z-projections of 27 one-micron-spaced confocal optical sections through the entire embryo, captured at 10-minute intervals. Scalebar = 10 μm.

Despite evidence that the identified motifs are required for the full fusogenic function of EFF-1 and an observation that a 14-3-3 protein can bind EFF-1 only in the presence of these motifs, there have been no data reported assessing whether *C*. *elegans* 14-3-3 proteins, FTT-2 and PAR-5, are needed for embryonic fusion events. We therefore examined 14-3-3 loss-of-function mutants for absence or aberrations of cell fusions in EFF-1-expressing hypodermal cells. Cell fusions were monitored during two major steps of cell fusion: 1) fusion pore formation indicated by diffusive cytoplasmic content mixing using the *elt-3p*::*yfp* reporter [70], a diffuse cytoplasmic, hypodermis specific reporter characterized here for the first time as a cell-cell fusion reporter (Fig 3 and S4 Movie) or *lbp-1p*::*gfp*, which shows similar hypodermis specific expression and cytoplasmic diffusion as *elt-3p*::*yfp* [36,51,76], and 2) widening of the fusion aperture seen by displacement and disappearance of intercellular junctions between fused cells using the AJM-1::GFP marker, a sub-adherens junction marker previously established as a cell-cell fusion reporter (Fig 4) [36,69,75]. These hallmark processes of cell fusion, cytoplasmic diffusion and disappearance of intercellular junctions, are delayed or absent in *eff-1* fusion mutants (Fig 3 lower panel and Fig 4 inset respectively). Transgenic 14-3-3 loss-of-function embryos expressing these cell fusion reporters were imaged throughout embryonic development and analyzed for defects in cell-cell fusions responsible for formation of the large hypodermal syncytium, hyp7.

**Fig 5:**
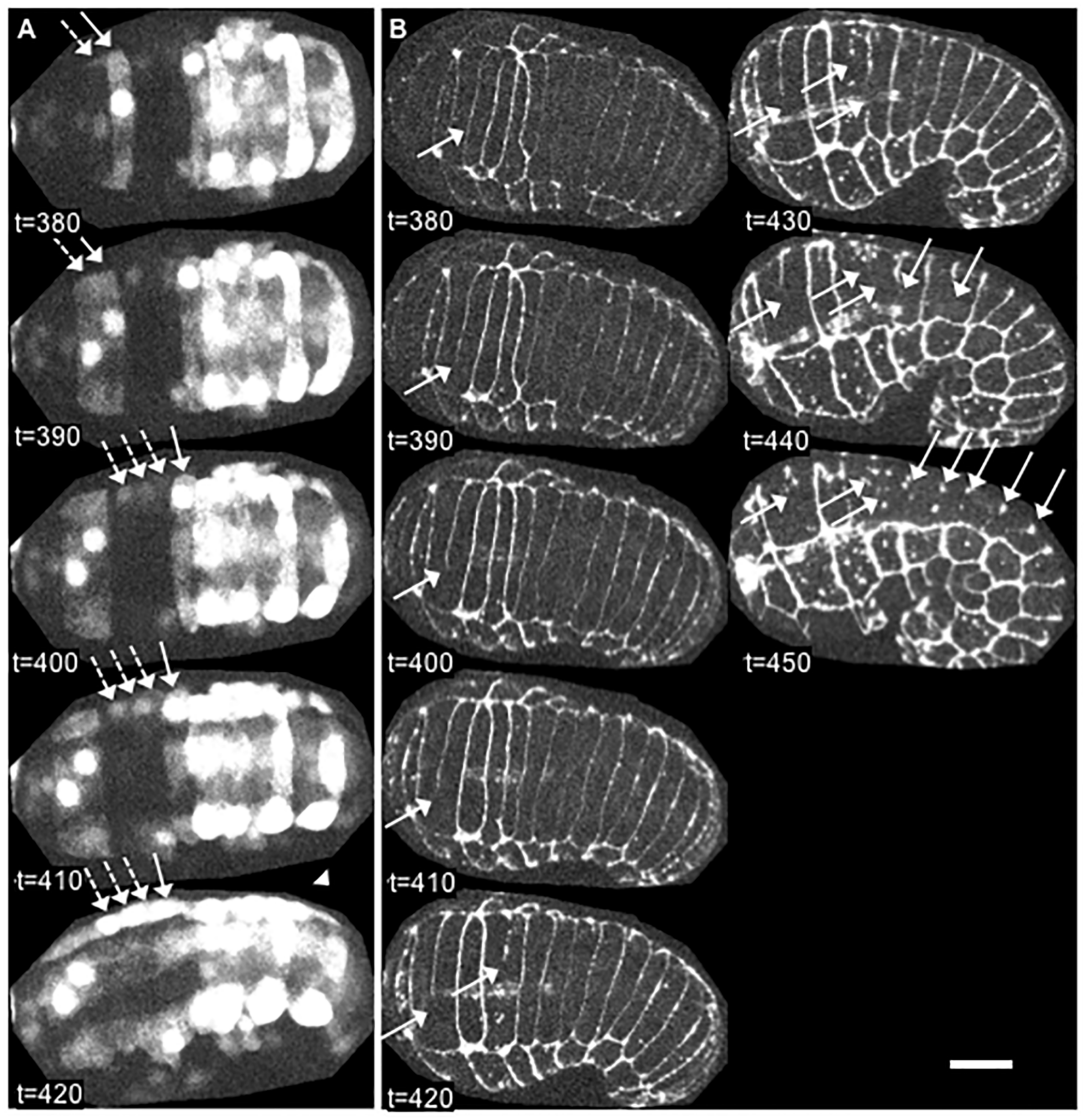
Cell-cell fusions in the absence of FTT-2. *Ftt-2* loss-of-function mutants show no disruption in the reproducible timing, position or orientation of cell-cell fusions in the developing epidermis. (A) Time-lapse images of a *ftt-2(n4426Δ)* null embryo expressing *elt-3p*::*yfp* show a hallmark of cell-cell fusion, the diffusion of YFP from labeled cells (solid arrows) to neighboring unlabeled cells (dashed arrows). Pattern observed in 100% of embryos (n = 5). (B) Time-lapse images of a *ftt-2* null embryo expressing a sub-adherens junction marker, AJM-1::GFP, show the disappearance of borders between fused cells (arrows). Pattern observed in 100% of embryos (n = 2). Anterior is left and dorsal is facing the viewer (t = 380–420) or oriented up (t = 430–450). Images shown are maximum intensity Z-projections of 27 one-micron-spaced confocal optical sections through the entire embryo, captured at 10-minute intervals. Scalebar = 10 μm.

First, we observed that functionally null *ftt-2* (*n4426Δ)* mutants showed no disruptions in the reproducible timing, position, or orientation of cell fusions, when compared to wild-type embryos and worms [33,35,69]. Cytoplasmic mixing (Fig 5A and S5 Movie) and recession and dissolution of intercellular junctions (Fig 5B and S6 Movie) occurred between all dorsal fusion-competent cells within the normal time-span of development. No ectopic fusions were seen.

**Fig 6:**
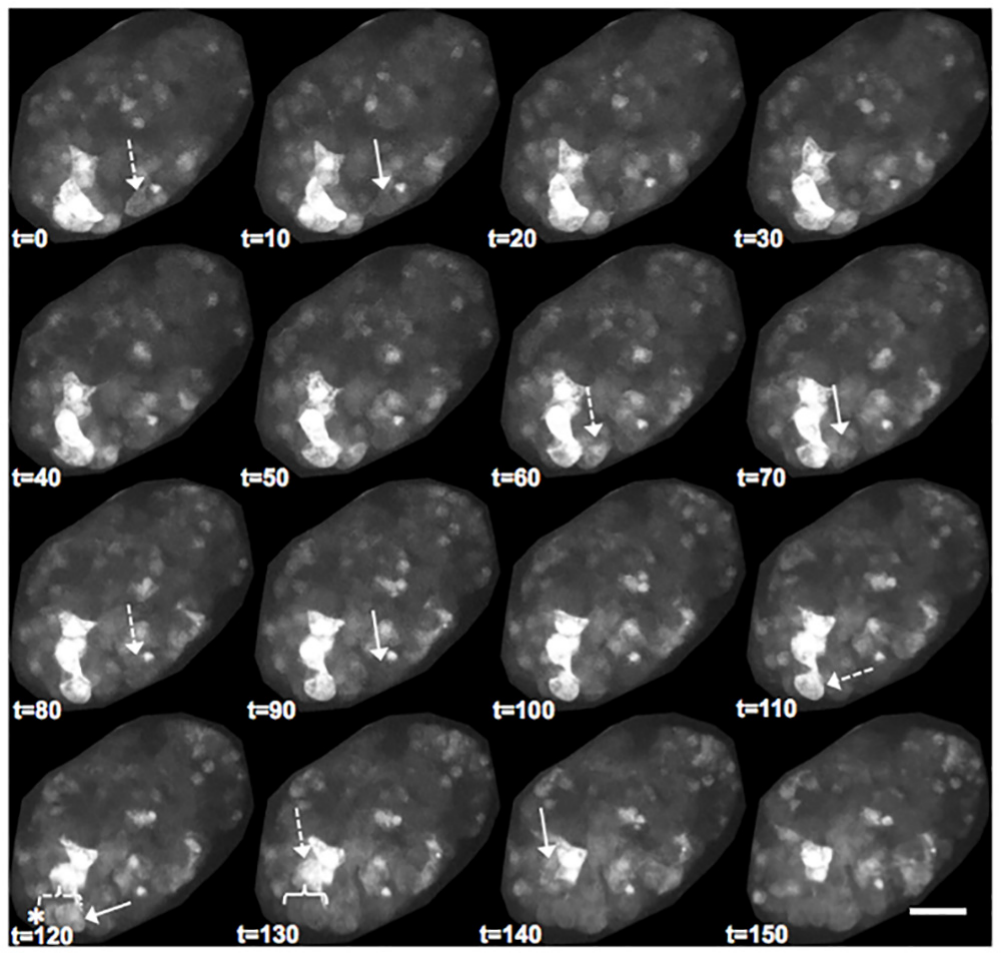
Cell-cell fusions occur in *par-5* loss-of-function mutants. Time-lapse images of a *par-5(it55)* embryo show multiple cell-cell fusion events, as seen in 100% of observed embryos (n = 3). A cytoplasmic reporter, *lbp-1p*::*gfp*, is specific to a subset of hypodermal cells that fuse to form hyp7. Three noticeable fusions events (t = 0–10, t = 60–70, t = 80–90) are apparent by a decrease in GFP fluorescence (solid arrow) of a brighter cell (dashed arrow) fusing with darker neighbors. Timepoints 110–130 reveal an adjacent bright cell (t = 110, dashed arrow) and dark cell that fuse with each other (t = 120, solid arrow and asterisk) to form a binucleated cell (dashed bracket). This binucleate cell subsequently dims while fusing with other neighboring cells (t = 130, solid bracket, decrease in fluorescence). Shortly after, one additional fusion event occurs (t = 130–140). Images shown are maximum intensity Z-projections of 27 one-micron-spaced confocal optical sections through the entire embryo, captured at 10-minute intervals. Posterior is lower-left and dorsal is lower-right. Scalebar = 10 μm.

Next, we assessed the requirement for PAR-5 in embryonic cell fusions. As previously reported, *par-5(it55)* animals exhibit the strongest expressivity compared to other characterized alleles and the overwhelming majority of *par-5(it55)* embryos fail to complete morphogenesis; however, cellular differentiation of multiple cell types does occur [63]. In our hands, homozygous *par-5(it55)* mutant hermaphrodites produced embryos that were embryonic lethal and characterized by failed morphogenesis. We assessed whether cell fusions occur in the resulting disordered tissues by use of the transgene *lbp-1p*::*gfp*, which is highly expressed in a subset of fusogenic cells of the hypodermal lineage. This reporter has previously been used to study hypodermal fusions by observing cytoplasmic diffusion of GFP between fusion-fated cells [36,51,76]. In *par-5* mutant embryos, we observed several cell-cell fusions taking place, even within aberrantly formed embryos. Fig 6 (S7 Movie) shows sets of neighboring bright and dark cells rapidly exchange fluorescent cytoplasm, a characteristic trait of cell fusions in these assays [36,38,51]. The fusion-competent cells seen in Fig 6 are most likely differentiated dorsal hypodermal cells (misshapen and poorly organized), because they display the strong fluorescence intensity of *lbp-1p*::*gfp* reporter expression that is typical of this cell type [76]. These results indicate that cell fusion is possible after loss of *par-5*, even while other aspects of hypodermal morphogenesis are severely affected.

### EFF-1 Induced Cell Fusions Persist After Reduction of Both 14-3-3 Paralogs

FTT-2 and PAR-5 are both expressed in the developing embryo [63,66]. Previous studies have shown that *ftt-2* mRNA expression more than doubles upon specific knockdown of *par-5* RNA, suggesting a possible compensation mechanism between *par-5* and *ftt-2* [72]. These data and the high sequence identity shared by PAR-5 and FTT-2 proteins (86.2%, [77]) puts forward the possibility that PAR-5 and FTT-2 could functionally compensate for one another in the single-mutant knockouts previously described. Consequently, a loss-of-fusion phenotype would not be seen in the single-mutant knockouts if either protein could regulate EFF-1 function. Accordingly, we reduced both PAR-5 and FTT-2 levels in developing embryos to examine the effect of loss of both species of 14-3-3 protein upon cell fusion.

In light of the diverse roles that 14-3-3s play in various cellular functions of eukaryotic organisms, it could be intractably lethal to completely eliminate all 14-3-3 proteins during *C*. *elegans* development. However, RNA interference in *C*. *elegans* allows genes to be knocked-down at different stages of development with adjustable expressivity. We therefore conducted double-knockdown experiments by inducing *par-5*-specific RNAi in *ftt-2* null hermaphrodites, using established dsRNA feeding techniques to induce systemic RNAi [78]. Initially, *par-5* RNAi was fed to *ftt-2(n4426Δ)* larvae beginning at the L1 stage; however, this resulted in potent sterility and gonad defects as the treated animals reached adulthood, a severe *par-5* mutant phenotype previously reported [63]. We subsequently adjusted the larval stages at which *par-5* RNAi was fed to *ftt-2* null larvae, to determine the developmental time period at which sufficient levels of maternal PAR-5 were expressed to allow for early gonad and oocyte development and the production of fertilized embryos. We found that feeding *ftt-2* null larvae with *par-5* dsRNA starting at the late-L2 to early-L3 stage (L2/L3) allowed for fertility in the treated larvae and avoided early stage maternal-effect embryonic lethality of their progeny. In this system, any hatching offspring from L2/L3 *par-5*-RNAi fed parents continued, themselves, to receive *par-5* RNAi through feeding during larval development post-hatching.

**Fig 7:**
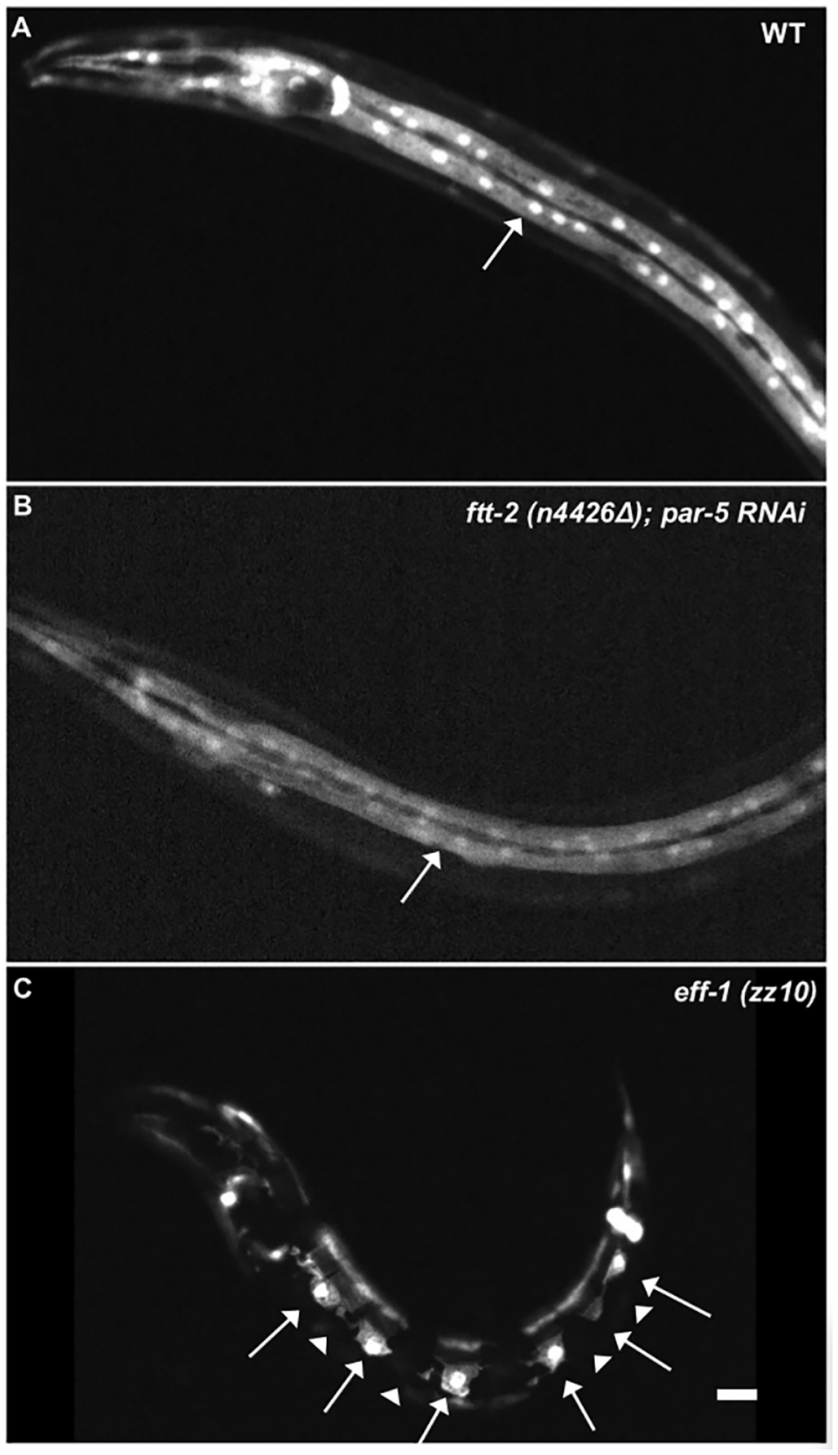
Larval hypodermal cell fusions occur normally in 14-3-3 double mutants. Double-knockdown mutant phenotype was generated using *par-5-*specific RNAi on *ftt-2(n4426Δ)* null mutant animals. Cells fated to fuse into the hyp7 syncytium of L4 larvae are labeled with *elt-3p*::*yfp* in three different genotypes: wild-type (A), 14-3-3 double knockdown (B), and *eff-1(zz10)* null mutant (C). In panels A and B, fields of syncytial hyp7 cytoplasm and nuclei display even and continuous distribution of YFP (arrows), as seen in 100% of observed larvae (n = 12 and 4 respectively). In the *eff-1* null larva in panel C, arrows indicate labeled hypodermal cells that have failed to fuse with hyp7 (arrowheads), as seen in in 100% of observed larvae (n = 6). Scalebar = 10 μm.

A strong *par-5* knockout phenotype was observed in these “escaping” progeny, as they showed gonad defects and sterility in adulthood. This *par-5* RNAi maternal effect phenotype corresponds with previous characterization of “escaping” progeny after RNAi [63]. Despite their fully expressed defects in gonadogenesis, however, these hatching offspring of *ftt-2-*null *par-5*-RNAi animals displayed no visible defects in hyp7 cell fusion, as seen in the pattern of *elt-3p*::*yfp* fluorescence, during their larval growth (Fig 7). Fig 7B shows a double row of hyp7 syncytial nuclei, resulting from embryonic and post-embryonic fusions, in a double-knockdown L4-stage larva. This pattern appears unperturbed when compared to a wild-type larva (Fig 7A) and contrasted with an *eff-1* null larva (Fig 7C), each also at the L4 stage. These results indicate that the four waves of larval hyp7 fusions occur correctly in the absence of *ftt-2* expression and with *par-5* expression as low as it can practically be suppressed. We concluded that these dozens of EFF-1-dependent hypodermal cell fusion events do not require normal levels of 14-3-3 proteins.

## Discussion

It is poorly understood how EFF-1 is regulated to accumulate and become actively fusogenic at the apical border of cells preparing for fusion. *In vivo* mosaic analyses by Podbilewicz *et al*. [41] and us (S2 Fig) show that fusion occurs only when both neighboring cells express EFF-1. This mutual dependence upon EFF-1 activity in adjacent fusion-fated cells invites a model of homotypic interaction between EFF-1 molecules at the interface between cell fusion partners [27,38,41,46]. As mentioned, recent structural studies of EFF-1’s ectodomain show homology to class II viral fusion proteins but with a unique mechanism from class II viral fusogens [45,46]. The current study explored possible interactions in *cis*, between EFF-1’s endodomain and other intracellular proteins that might regulate its function and localization. Our previous results [51] had offered seemingly strong evidence suggesting that 14-3-3 adaptor molecules bind to predicted 14-3-3 binding motifs in EFF-1’s cytoplasmic domain and thereby potentiate cell-fusion activity. From those preliminary results and the multiple cellular roles in which 14-3-3s engage, we modeled several hypotheses of how 14-3-3 could regulate EFF-1 at the level of localization, oligomerization, or the activation of fusogenicity.

Despite the earlier evidence that stimulated these hypotheses, however, the current study reveals that 14-3-3s are not key regulators, either spatially or temporally, of hypodermal cell fusion directed by EFF-1. We have shown here that EFF-1A::GFP is correctly translocated and retained at hypodermal cell membranes programmed to fuse, even when the possibility of 14-3-3 binding has been abrogated. Likewise, the EFF-1 cell fusion machinery is not affected by the loss of 14-3-3 proteins, as evidenced by the persistence of hypodermal cell-cell fusions in embryogenesis and larval development when PAR-5 and FTT-2 have been reduced or eliminated. However, we cannot exclude the possibility that EFF-1A may be regulated by 14-3-3s in other syncytial tissues or as part of distinct functions or mechanisms. The range of tissues and timing at which EFF-1 is thought to trigger membrane fusion is quite diverse [2,36,39,79,80]. We have not surveyed all of these instances for fidelity in either the rate or accuracy of fusion events. Nonetheless, in the visibly prominent cell fusions that we studied here, loss of potential interaction motifs from EFF-1 or loss of potential interacting proteins PAR-5 and FTT-2 did not induce any measurable deficit in protein localization or fusogenic activity of EFF-1, respectively.

### EFF-1’s Cytoplasmic Domain and Its Effect on Fusogenicity

It appears that the C-terminal cytoplasmic tail of the EFF-1A splice variant, harboring the putative 14-3-3-binding consensus motifs, is required for only some actions of the *eff-1* gene during development. The quantitative analysis from our high-resolution imaging of cell fusions in the embryo suggest that visible defects in the form of *eff-1(zz1)* larvae may result from slight delays in fusions that are critically important to formation of the tail-whip structure. It was recently shown that AFF-1, but not EFF-1, is necessary for tail-spike cell fusion [81,82]. Therefore, the actions of EFF-1 function must be necessary for other events required for normal tail-whip formation, perhaps hyp10 cell fusion, considering the highly penetrant tail-whip defects seen in *eff-1(zz1)* and all other currently characterized *eff-1* mutants. Our new data combine with previous descriptions of abnormal tail-whip morphogenesis to suggest that the highly penetrant larval tail-whip defects seen in *eff-1(zz1)* are likely due to a rather short delay in cell fusions. It seems, therefore, that the morphogenesis of some structures, such as the tail, resulting from EFF-1 function are more exacting in their need for temporally precise fusion events than are other structures in the body plan. Alternatively, it is possible that other non-tail tissues simply suffer less-visible or less-functionally obvious defects when EFF-1-dependent fusions are slightly delayed by a minor decrease in EFF-1 activity. It may also be true that the EFF-1A isoform is uniquely required for tail-whip morphogenesis, the only context in which the functions of EFF-1A and -1B have been compared directly *in vivo* [38]. These possibilities remain to be tested.

We hypothesize that EFF-1(*zz1*)’s decreased potency is a result of decreased protein stability, impairment of cell surface expression, or fusogen activation. Reduced viral fusogen oligomerization and reduced interaction of viral fusogens with accessory proteins at the membrane have both been reported in viral fusion-protein mutants with truncated cytoplasmic tails [49,83]. We can exclude the possibility that EFF-1A’s cytoplasmic tail is essential to the core membrane fusion mechanism, because cell fusions in *eff-1(zz1)* do occur, albeit at a slightly delayed rate. But the possibility remains that the EFF-1A C-terminus offers a fusion-enhancing function, as has been reported for some viral fusogens. For example, truncation of the simian parainfluenza virus 5 (SV5) fusion protein’s cytoplasmic domain impedes fusion pore enlargement and the endodomain of reovirus FAST fusogens is important for the membrane fusion mechanism and syncytiogenesis [50,84]. Future studies will be needed to determine whether the fusogenic activity per EFF-1 molecule is actually affected by this or any mutation. Currently, our data do not discriminate between changes in stability, activity, or localization.

### Comparing Analyses of EFF-1 Function *in vivo* and in Exogenous Assay Systems

This report describes techniques that more precisely measure fusogen function potency in worm strains carrying modified versions of *eff-1*. Fine tail whip structure remains the most sensitive bioassay for the detection of a slight reduction in *eff-1* function, as it is disrupted in all alleles that we have studied. The strong penetrance of tail defects produces an essentially binary signal. Defects are seen in the tail whip of all offspring with reduced or absent EFF-1 activity versus a normal tail whip phenotype in all offspring of wild-type EFF-1 animals. Alleles with severe loss of molecular integrity present with defects in body length and morphology. Analysis of a range of mutant alleles using these assays allows us to rank-order molecular defects by the degree of decreased body length (Fig 1A). In contrast, the embryonic hyp7 fusion-timing assay—used here to measure deficits in the *eff-1(zz1)* mutant (Fig 1B and 1C)–should allow for quantification of subtle differences in fusogenic activity among weakly hypomorphic or hypermorphic *eff-1* alleles. Fine tail whip structure, body length, and hyp7 fusion-timing could be used to assess the activity of transgenic EFF-1 variants expressed during mutant-rescue assays but the strength of EFF-1 activity produced from endogenous and transgene loci has yet to be quantitatively reconciled for *eff-1*. Until reconciled, direct evaluative comparisons between mutations *in loco* and *in vitro* are not possible.

Concordance between heterologous organism- and cell-based assays, *in vivo* assays, and predictive algorithms varies. We found in our analysis of *eff-1(zz1)* that the loss of the full EFF-1A C-terminal tail produces a measurable deficit in the fusogenic activity of *eff-1* gene products *in vivo*. These conclusions agree with data previously published using transfected EFF-1A and EFF-1B cDNAs to induce fusion of cultured insect cells [41]. Thus, some structure/function analyses carried out in an alternative experimental system can correctly predict the behavior of the molecule within the cells of developing nematode tissues. In contrast, we saw no appreciable impact on EFF-1 localization or fusogenic function when we examined mutations specifically disrupting a predicted interaction between EFF-1 and endogenous 14-3-3 proteins. In this case, our observations do not harmonize with previous evidence or predicted function for a physical binding interaction between EFF-1A and a human 14-3-3 protein. Apparently, in this case, the extrapolations from *in vitro* data are not supported *in vivo*. Alternatively, the impact of the loss of a 14-3-3/EFF-1 interaction, if one exists *in vivo*, must be slight or difficult to visualize in the tissues we examined.

### Post-Translational Modification of the Cytoplasmic Domain of EFF-1

Our previous results showed that alanine-substitution of serines S632, S634, and S654 reduces the activity of EFF-1 (expressed from a transgene) and blocks the ability to rescue endogenous mutations [51]. While we have not directly shown that these serines are phosphorylated *in vivo*, our previous *in vitro* results showed that a phospho-specific protein, human 14-3-3, will only bind EFF-1A::GFP if these three serine residues have not been replaced with non-phosphorylatable alanines. In addition, threonine-substitution of these serines, to retain a phosphorylatable residue at each position, conserves the cell-fusion activity of an *eff-1* transgene expressed in the nematode [51]. The NetPhos 2.0 phosphorylation prediction algorithm scores S632 and S634 as highly likely to be phosphorylated, and S654 as not as likely [85]. Interestingly, site-directed deletion of S654 (653–655) from EFF-1A has no detrimental effect on a transgene’s ability to rescue *eff-1(oj55)* mutants (data not shown). For reasons we cannot explain, it seems that mutation of the S654 to alanine proves to be more detrimental than this triplet deletion. The strong prediction of S632 and S634 phosphorylation gives reason to believe that these serines on the EFF-1A C-terminus may become phosphorylated in *C*. *elegans in vivo*.

Using a prediction tool (KinasePhos 2.0) for phosphorylation sites and the kinases that act on such sites, we found that multiple kinases are predicted to act on S632, S634, and S654 [86]. Testing whether EFF-1-dependent cell fusion is sensitive to the activity of *C*. *elegans* kinase homologues may be an interesting course of investigation as control of EFF-1 fusogenicity by phosphorylation would be a novel form of fusogen regulation. Likewise, generation of phosphomimetic mutations (aspartate or glutamate) at S632 and S634 might reveal phosphorylation effects on EFF-1 function that are detectable by one of the *in vivo* assays established in this study. To our knowledge, phosphorylation has been shown to only indirectly affect virus-cell fusion and virally induced syncytium formation [87,88]. In any hypothesis, modification of EFF-1A’s cytoplasmic tail can only modulate its activity to generate efficiently timed cell fusions, since we have shown here that delayed EFF-1-dependent fusions can still occur in the absence of its C-terminus.

## Supporting Information

S1 Fig Full-length EFF-1A protein sequence. The cytosolic domain is underlined, the location of the premature stop in *eff-1(zz1)* is marked with a box, and the 14-3-3 motifs are in bold with the key residues highlighted gray.
(TIF)

S2 Fig Mosaic strain analysis confirms mutual requirement for EFF-1 in both fusing cells. Cells without DsRed2 have lost a rescuing *eff-1* transgene and have reverted to an *eff-1(zz10)* null genotype. Cell borders are highlighted by AJM-1::GFP. Images are projections of stacks of confocal optical sections. In all panels, dorsal is up, ventral is down. (A) A larva with uniform hypodermal expression of DsRed2 is completely rescued for dorsal hyp7 cell fusion. There are no remaining unfused dorsal junctions in hyp7. (B-D) Three examples of mosaic worms with unfused rescued/mutant cell pairs. In (B) and (C), filled arrowheads show unfused borders between cells. (D) Top panel, AJM-1::GFP localization showing intact junctions (green hollow arrowheads); middle panel, DsRed2 expression, indicating *eff-1+* genotype, with the number of red cells fused into each small syncytium indicated (red dotted lines); bottom panel, merged image showing *eff-1* null cells (white arrowheads) separated from fused *eff-1+* neighbors by intercellular AJM-1::GFP junctions. In contrast to these examples, we found only one instance (out of 768 fusion-fated cell borders assayed) of an unfused cell junction lying between pairs of DsRed2-positive *eff-1+* cells. Although this rare cell pair may have expressed levels of exogenous EFF-1 insufficient to elicit timely cell fusion, the observed 99.87% efficiency of fusion in cases of mutual EFF-1 expression underscores the repeated failure to fuse of cell pairs mismatched for EFF-1 expression. These results agree with those of Podbilewicz et al. in cultured cells and in similarly generated mosaic animals [41], and therefore strongly support the model that EFF-1 acts homotypically, required by both cells for fusion to occur. **Strain Construction:** FC196: N2 (Bristol) *C*. *elegans* hermaphrodites were transformed by microinjection of pSur5Rc and pJE8 (wild-type *eff-1*) to generate extrachromosomal array *zzEx78*. pSur5Rc, a gift from Morgan Tucker and Min Han at the University of Colorado, includes the DsRed2 coding region (Clonetech) ligated via KpnI/EcoRI subcloning downstream of a 3.6 kb *sur-5* promoter, originally derived from pTG96.2 [89]. Worms with red nuclear fluorescence were selected from the progeny following injection and were crossed to N2 males. FC204: FC196 (*zzEx78*[*eff-1+; pSur5Rc*]) males were mated with FC75 (*eff-1(zz10) II; jcIs1 IV*) hermaphrodites. A strain carrying *zzEx78* and homozygous for both *eff-1(zz10)*, and *jcIs1* was identified by observing that all worms not carrying *zzEx78* exhibited 100% fusion-defective phenotypes (homozygous *eff-1(zz10)*), while progeny carrying *zzEx78* were rescued for larval tail-whip defects and disappearance of AJM-1::GFP junctions in the hypodermis. **Imaging:** Loss of the extrachromosomal array *zzEx78* expressing *eff-1+*and DsRed2 during embryonic cell division results in a mosaic pattern of red fluorescence. Mosaic animals were identified in which *eff-1*-rescued cells expressing DsRed2 lay adjacent to non-red (*eff-1* null) cells. Larvae were paralyzed with 1M sodium azide and confocal image stacks were acquired on either a Perkin Elmer Ultraview RS5 or a Zeiss LSM 510 Meta confocal scanning microscope. Laser excitation used was at 488nm for GFP excitation and either at 568nm or at 543nm for DsRed2. GFP and DsRed2 channels were separated using linear unmixing software (Zeiss). Confocal z-stacks were converted to TIFF format and rendered as projections using Image J software [74].
(TIF)

S1 Movie Animals expressing EFF-1A with a C-terminal truncation have delayed embryonic cell fusions. Maximum-intensity projection of an *eff-1(zz1)* embryo expressing an adherens junction marker (AJM-1::GFP) imaged by 4-dimensional confocal microscopy. Arrows denote fused junctions and arrowheads indicate unfused cell borders, with intact junctions still observed before the embryo begins muscular movement. Anterior is left, dorsal is up. Time shown is approximate age since fertilization. Scalebar = 10 μm. Early cytoplasmic fluorescence seen in gut-fated cells (no longer visible at the time of adherens junctions phenotyping) is expressed from the mIs12 transgene, which was included in the background in which we screened for the zz1 mutation and is tightly linked to *eff-1* on chromosome II.
(MOV)

S2 Movie EFF-1(S632/634/654A)::GFP accumulation at a fusion-fated cell border on the ventral embryo surface. Time-lapse maximum-intensity projection of the ventral surface of the embryo shown in Fig 2A. Arrow indicates EFF-1(S632/634/654A)::GFP accumulation at the cell contact. Scalebar = 10 μm.
(MOV)

S3 Movie EFF-1(S632/634/654A)::GFP accumulation at a fusion-fated cell border on the dorsal embryo surface. Time-lapse maximum-intensity projection of the dorsal surface of the embryo. Arrow indicates EFF-1(S632/634/654A)::GFP accumulation at a cell contact. One-micron-spaced image stacks were captured every 2.5 minutes using widefield microscopy, and maximum intensity Z-projections of the dorsal surface were rendered. In 100% of the mutant embryos (n = 4), the same pattern of junctional localization is seen as for wild-type EFF-1::GFP [38]. Scalebar = 10 μm.
(MOV)

S4 Movie Cell fusions in an embryo expressing *elt-3p*::*yfp*, a cytoplasmic hypodermis-specific reporter. Time-lapse maximum-intensity projection of the wild-type embryo shown in Fig 3. Arrows denote the anterior and posterior limits of each successively expanded multinucleated cell during the stepwise fusion events that create the large hyp6 and hyp7 syncytia. Yellow arrows show specific fusion events that were monitored in optical-section time-lapse recordings of mutant and rescued genotypes in Figs 5 and 7. Scalebar = 5 μm.
(MOV)

S5 Movie Cell fusions in an *ftt-2(n4426Δ)* null embryo expressing *elt-3p*::*yfp*, a cytoplasmic hypodermis-specific reporter. Time-lapse maximum-intensity projection of the embryo shown in Fig 5A. White arrows indicate bright cells prior to fusion with neighboring dark cells. Yellow arrows show syncytial cells after equilibration of cytoplasmic fluorescence. Scalebar = 10 μm.
(MOV)

S6 Movie Cell fusions in an *ftt-2(n4426Δ)* null embryo expressing the intercellular junction marker AJM-1::GFP. Time-lapse maximum-intensity projection of the embryo shown in Fig 5B. White arrows indicate disappearing junctions between fusing cells. Scalebar = 10 μm.
(MOV)

S7 Movie Cell fusions in a *par-5(it55)* loss-of-function embryo expressing *lbp-1p*::*gfp*, a cytoplasmic hypodermis-specific reporter. Time-lapse maximum-intensity projection of the embryo in Fig 6. White arrows indicate bright cells prior to fusion with neighboring cells. Yellow arrows indicates cell fusion event observed by a decrease in reporter expression after fusion. Red arrow denotes a bi-nucleated cell. Scalebar = 10 μm.
(MOV)

S1 SUPPORTING INFORMATION Nonsense mediated decay (NMD) assay. (DOCX)
