# Supplementary figures and images for: The EFF-1A Cytoplasmic Domain Influences Hypodermal Cell Fusions in C. elegans But Is Not Dependent on 14-3-3 Proteins
Source: PLoS One. 2016 Jan 22;11(1):e0146874. doi: 10.1371/journal.pone.0146874 (PMC4723337; doi:10.1371/journal.pone.0146874)

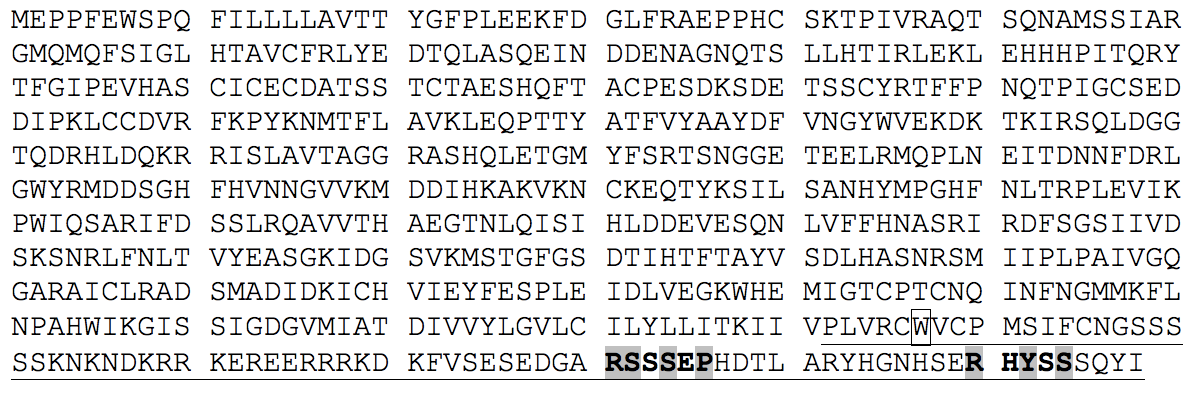

Supplement: S1 Fig — The cytosolic domain is underlined, the location of the premature stop in eff-1(zz1) is marked with a box, and the 14-3-3 motifs are in bold with the key residues highlighted gray. (TIF) [file pone.0146874.s001.tif]

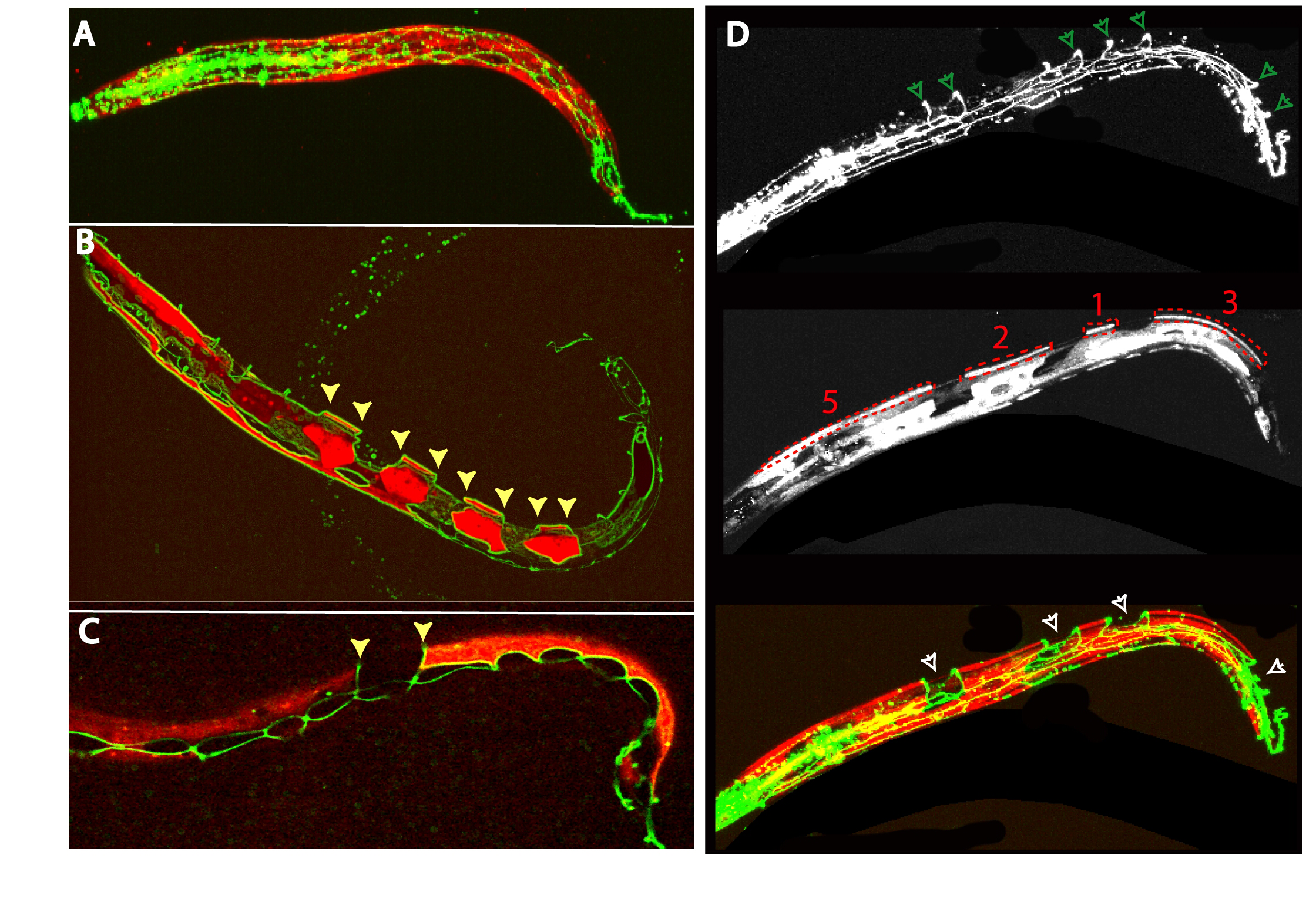

Supplement: S2 Fig — Cells without DsRed2 have lost a rescuing eff-1 transgene and have reverted to an eff-1(zz10) null genotype. Cell borders are highlighted by AJM-1::GFP. Images are projections of stacks of confocal optical sections. In all panels, dorsal is up, ventral is down. (A) A larva with uniform hypodermal expression of DsRed2 is completely rescued for dorsal hyp7 cell fusion. There are no remaining unfused dorsal junctions in hyp7. (B-D) Three examples of mosaic worms with unfused rescued/mutant cell pairs. In (B) and (C), filled arrowheads show unfused borders between cells. (D) Top panel, AJM-1::GFP localization showing intact junctions (green hollow arrowheads); middle panel, DsRed2 expression, indicating eff-1+ genotype, with the number of red cells fused into each small syncytium indicated (red dotted lines); bottom panel, merged image showing eff-1 null cells (white arrowheads) separated from fused eff-1+ neighbors by intercellular AJM-1::GFP junctions. In contrast to these examples, we found only one instance (out of 768 fusion-fated cell borders assayed) of an unfused cell junction lying between pairs of DsRed2-positive eff-1+ cells. Although this rare cell pair may have expressed levels of exogenous EFF-1 insufficient to elicit timely cell fusion, the observed 99.87% efficiency of fusion in cases of mutual EFF-1 expression underscores the repeated failure to fuse of cell pairs mismatched for EFF-1 expression. These results agree with those of Podbilewicz et al. in cultured cells and in similarly generated mosaic animals [41], and therefore strongly support the model that EFF-1 acts homotypically, required by both cells for fusion to occur. Strain Construction: FC196: N2 (Bristol) C. elegans hermaphrodites were transformed by microinjection of pSur5Rc and pJE8 (wild-type eff-1) to generate extrachromosomal array zzEx78. pSur5Rc, a gift from Morgan Tucker and Min Han at the University of Colorado, includes the DsRed2 coding region (Clonetech) ligated via KpnI/EcoRI su [file pone.0146874.s002.tif]
