# Supplementary material for: The EFF-1A Cytoplasmic Domain Influences Hypodermal Cell Fusions in C. elegans But Is Not Dependent on 14-3-3 Proteins
Source: PLoS One. 2016 Jan 22;11(1):e0146874. doi: 10.1371/journal.pone.0146874 (PMC4723337; doi:10.1371/journal.pone.0146874)
Supplement: S1 SUPPORTING INFORMATION — (DOCX) [file pone.0146874.s010.docx]

**S1 Supporting Information. Nonsense mediated decay (NMD) assay.**

Strain FC57 (*eff-1(zz1); mIs12 II*) was crossed to PD8117 (*smg-1(cc545) unc-54(r293) I*). SMG-1 is a protein kinase essential for NMD in *C. elegans* [1]. The *unc-54(r293)* allele encodes a premature termination codon, which targets the message for degradation by NMD at permissive temperature (16°C), resulting in uncoordinated movement (Unc). At restrictive temperature (25°C), SMG-1(cc545) is inactivated and the Unc phenotype is partially suppressed. Triple mutant cultures (*eff-1 (zz1), smg-1(cc545), unc-54(r293)*) were maintained for several days at both 16°C and 25°C conditions*.* Adults were confirmed for Unc phenotype, and larvae were observed for Eff phenotypes in tail whip morphology. We confirmed that the NMD-dependent Unc-phenotype was readily suppressed at 25°C in e*ff-1(zz1), smg-1(cc545)*, *unc-54(r293)* triple mutants, indicating inactivation of NMD at this temperature. However, this non-permissive state for NMD failed to reconstitute the Eff mutant larval tail whip, a phenotype that remained defective in all screened triple mutants at 25°C (n>100). Triple mutants also retained Eff phenotype at NMD-permissive temperatures (n>100). Our observations of no change in tail phenotype at either temperature suggest that loss of function in *eff-1(zz1)* is due to defects within the expressed protein, rather than NMD degradation of the message brought on by a nonsense mutation.

# References

1. Grimson A, O’Connor S, Newman CL, Anderson P (2004) SMG-1 is a phosphatidylinositol kinase-related protein kinase required for nonsense-mediated mRNA Decay in Caenorhabditis elegans. Mol Cell Biol 24: 7483-7490.
